# A Platform for Rapid, Quantitative Assessment of Multiple Drug Combinations Simultaneously in Solid Tumors *In Vivo*

**DOI:** 10.1371/journal.pone.0158617

**Published:** 2016-06-30

**Authors:** Joyoti Dey, William S. Kerwin, Marc O. Grenley, Joseph R. Casalini, Ilona Tretyak, Sally H. Ditzler, Derek J. Thirstrup, Jason P. Frazier, Daniel W. Pierce, Michael Carleton, Richard A. Klinghoffer

**Affiliations:** 1 Presage Biosciences, Inc., Seattle, Washington, United States of America; 2 Celgene Corporation, San Francisco, California, United States of America; Barts Cancer Institute, UNITED KINGDOM

## Abstract

While advances in high-throughput screening have resulted in increased ability to identify synergistic anti-cancer drug combinations, validation of drug synergy in the *in vivo* setting and prioritization of combinations for clinical development remain low-throughput and resource intensive. Furthermore, there is currently no viable method for prospectively assessing drug synergy directly in human patients in order to potentially tailor therapies. To address these issues we have employed the previously described CIVO platform and developed a quantitative approach for investigating multiple combination hypotheses simultaneously in single living tumors. This platform provides a rapid, quantitative and cost effective approach to compare and prioritize drug combinations based on evidence of synergistic tumor cell killing in the live tumor context. Using a gemcitabine resistant model of pancreatic cancer, we efficiently investigated nine rationally selected Abraxane-based combinations employing only 19 xenografted mice. Among the drugs tested, the BCL2/BCLxL inhibitor ABT-263 was identified as the one agent that synergized with Abraxane^®^ to enhance acute induction of localized apoptosis in this model of human pancreatic cancer. Importantly, results obtained with CIVO accurately predicted the outcome of systemic dosing studies in the same model where superior tumor regression induced by the Abraxane/ABT-263 combination was observed compared to that induced by either single agent. This supports expanded use of CIVO as an *in vivo* platform for expedited *in vivo* drug combination validation and sets the stage for performing toxicity-sparing drug combination studies directly in cancer patients with solid malignancies.

## Introduction

While the past several decades of cancer research have led to a vastly increased understanding of the complex mechanisms that underlie cancer cell survival, particularly in the face of stressors such as chemotherapy, the failure rate of new oncology drugs in clinical trials compared to other disease areas is still exceedingly high (roughly 85%, [1]). This reality has served to mobilize efforts directed at developing combination therapies that directly inhibit, or prevent development of, drug resistance in cancer patients. Indeed, based on these efforts, we are now beginning to observe increased therapeutic benefit for patients with certain malignancies, highlighted by the prolonged survival of melanoma patients receiving a combination of dabrafenib (BRAF inhibitor) and trametinib (MEK inhibitor) [2, 3]. Unfortunately as seen with single agent trials, clinical successes for most novel drug combinations are rare, highlighting inefficient translation from the laboratory to the clinical setting [4].

Discovery, validation, and prioritization of effective synergistic drug combinations for pursuit in the clinic is a factorial problem that is not efficiently addressed by current methods of *in vivo* analysis in the preclinical setting. Statistically thorough quantification of synergy using intact *in vivo* tumor models is resource intensive and time consuming, particularly when an investigator desires to compare multiple drug combinations of interest simultaneously. Barriers to *in vivo* anti-cancer drug combination analysis include, but are not limited to requirements for scale-up of sufficient quantities of compound for *in vivo* dosing, establishment of proper dosing regimens to avoid toxicity and legitimately evaluate combination effects, and large numbers of tumor-bearing animals to achieve study significance. Furthermore, statistically rigorous assessment of cancer drug synergy, even for a simple two-compound drug combination, in the context of the human clinic is not currently feasible. Therefore, a need exists for a methodology that incorporates a multiplexed framework for statistically valid drug combination analysis, but importantly is adapted to a relevant *in vivo* system that recapitulates the dynamic genotypic and phenotypic heterogeneity of a tumor in its microenvironment.

We therefore sought to develop an approach to *in vivo* cancer drug combination analysis that is reproducible, quantitative and statistically rigorous, feasible for incorporation into existing drug development undertakings including human clinical trials and most notably, predictive of long term systemic outcomes. Towards this goal, we adapted the previously described CIVO microinjection platform, which enables assessment of multiple drugs simultaneously in single living solid tumors, to *in vivo*, in-tumor investigation of drug combinations. First, we optimized CIVO to perform head-to-head comparisons of a panel of possible combination therapies to select the best candidate(s) for further investigation. Second, we combined CIVO with a statistically rigorous model of combination effects to test specific combination therapies for synergistic anti-tumor effects.

In this study, we demonstrate the potential of these methods through a focused study in a preclinical model of gemcitabine-refractory pancreatic cancer. Specifically, the CIVO platform was used to identify agents that enhance the efficacy of Abraxane^®^ (nab paclitaxel) and thus ultimately provide a rational alternative to gemcitabine in patients who are resistant to treatment with this drug. Of the nine combinations analyzed with our method, the combination of Abraxane^®^ with the BCL2/BCLxL inhibitor ABT-263 was found to induce synergistic pancreatic tumor cell apoptosis, a result which was verified upon systemic administration of these agents in an independent pre-clinical combination study.

## Materials and Methods

### Reagents

Compounds used in the CIVO and systemic studies were purchased from Selleck Chemicals (Everolimus, Sunitinib, 5-Fluorouracil, Rapamycin, Gemcitabine, ABT-199, ABT-263, Erlotinib), Chemietek (ABT-263, ABT-199) and Medkoo Biosciences (Mitomycin C). Abraxane^®^ was manufactured by Celgene Corporation (San Diego, CA).

### Cell culture

Mia PaCa2 pancreatic cancer cell line (ATCC) were cultured in DMEM (ThermoFisher) plus 10% fetal bovine serum. Cultures were grown at 37°C, 5% CO2.

### *In vivo* studies

All work in mice was approved by the respective IACUC Boards of the Fred Hutchinson Cancer Research Center, Seattle, WA and Presage Biosciences, Seattle, WA. All relevant procedures were performed under anesthesia and all efforts were made to minimize pain and suffering. None of the mice contributing to this study became ill or died prior to experimental endpoints and all mice receiving drug treatment as described below, underwent routine health monitoring and were humanely euthanized at the end of the experiments. Subcutaneous flank xenografts were generated in athymic nude mice (Harlan Laboratories) using the MiaPaCa2 cells inoculated at 5x10^6^ million cells per mouse as per protocol described in [5]. Mice were enrolled into CIVO drug combination studies when the tumor volume reached approximately 1000 mm^3^. At this size, tumors exhibited multiple aspects of a heterogeneous microenvironment including regions of vascularization and hypoxia, collagenous extracellular matrix, vascular endothelial cells, and infiltrating macrophages (S1 Fig). Microinjection studies were performed using the CIVO device as previously described [5]. The device was configured with 6 injection needles set for a 6 mm injection length and a total volume delivery of 3 μl. Because previous studies including radiolabeled drug injections [5] have shown that drug concentrations are undetectable beyond 1.5 mm from the site of injections, needles were separated by a minimum distance of 3 mm. A fluorescent tracking marker (FTM) was added to each drug reservoir in vehicle for delivery along with each drug or drug combination. All micro-doses were equivalent to or lower than what would be allowed under FDA guidelines for Exploratory IND (Investigational New Drug) studies and by solubility of drug into vehicle. Total amounts of agents injected were—Abraxane^®^ 14.2 μg; Mitomycin C 2.5 μg; Everolimus 7.2 μg; Sunitinib 3.0 μg; 5-Fluorouracil 975 ng; Rapamycin 6.9 μg; Gemcitabine 2.0 μg; ABT-199 6.5 μg; ABT-263 7.3 μg and Erlotinib 3.0 μg. Tumors were resected from euthanized mice 24 hours after microinjection for immunohistochemical analysis.

For systemic drug efficacy studies, Abraxane^®^ was formulated in 0.9% saline and administered intravenously at 10, 20 or 30 mpk; ABT-263 was formulated in 60% Phosal 50, 30% PEG400 and 10% ethanol and administered by oral gavage at 100 mpk. Mice were enrolled for study when tumors reached a volume of 200 mm^3^. Tumor volume was calculated as *V* = length x width x height, all three dimensions measured using digital calipers [6]. Study endpoint was defined as when an animal had to be removed from the study due to any one of the three measured dimensions of the tumor exceeding 2 cm or body weight loss greater than 20%. Tumor Growth Inhibition % (TGI) is defined as

$$\frac{\left(V_{final}\left(\text{vehicle}\right)-V_{initial}\left(\text{vehicle}\right)\right)-\left(V_{final}\left(\text{treatment}\right)-V_{initial}\left(\text{treatment}\right)\right)}{\left(V_{final}\left(\text{vehicle}\right)-V_{initial}\left(\text{vehicle}\right)\right)}\times 100$$

where measurements are averaged across tumors in respective arms. Wilcoxon Rank Sum test was used as the statistical test to determine differences between treatment arms (n = 8 per arm)

### Tissue processing, gross tissue imaging and immunohistochemistry (IHC)

Following tumor resections, 2 mm thick sections perpendicular to the injection columns, were fixed in 10% buffered formalin for 48 hours, scanned and processed as previously described [5]. Rabbit anti-CC3 antibody (Cell Signaling, 1:150 dilution), mouse anti pHH3 (Cell Signaling, 1:200 dilution), mouse anti-GLUT1 (Abcam ab40084, 1:250 dilution), rabbit anti-CD31 (Abcam ab28364, 1:250 dilution), rabbit anti-SMA (Dako M0851, 1:1000 dilution), rabbit anti-S100A9 (Abcam 63818, 1:5000 dilution) were used for IHC analysis. For immunofluorescent detection, secondary antibodies conjugated to AlexaFluor647 (Jackson Immunoresearch, 1:600 dilution) and AlexaFluor555 (Invitrogen, 1:500 dilution) was applied according to manufacturer’s instructions and tissues counterstained with DAPI.

### Measurement of drug response

Quantitative analysis of IHC was performed using custom software [5] (CIVOanalyzer; Presage Biosciences, Seattle). Within this package injection sites are automatically detected via the fluorescent tracking markers. Circular regions of interest (ROIs) centered on the injection sites are inscribed. Within these ROIs, the fractions of cells affected (e.g. CC3 positive) are mapped as a function of radial distance in 100 μm increments from the injection site. This is referred to as the radial effect curve *f*(*r*). To mitigate the influence of pre-existing necrosis on these measurements, injection sites that fall within largely acellular tumor regions are excluded prior to quantitative analysis.

### Statistical approach for multi-drug combination analysis

The multiplexed design of CIVO enables head-to-head comparison of different injected compounds in the same tumor. In the case of combinations, this feature can be used to measure the increase in response generated when a second compound is added to a primary compound. By comparing the increase in response of the second agent paired with multiple different primary compounds, the pair that generates the largest increase can be identified and considered to be the best candidate for synergy analysis.

In this investigation, Abraxane^®^ was paired with each of the 9 compounds listed in Table 1. For each compound, the change in response due to addition of Abraxane^®^ was estimated at each radial distance and for the integrated area under this curve using linear mixed effects models [7]. In the models, the Abraxane^®^ response was treated as a fixed effect and the response to the primary agent was treated as a random, tumor-dependent effect. Each model was generated using R version 3.0.2 (The R Foundation for Statistical Computing) with the lme4 package for linear mixed models.

**Table 1:** CIVO screen for drugs that are synergistic with pancreatic cancer standard of care Abraxane^®^ in a Gemcitabine resistant xenograft model.

Drug	Mechanism of Action
Abraxane	Albumin bound anti mitotic agent
Fluorouracil (5FU)	Nucleoside analog inhibiting DNA replication and repair
ABT-199	Selection inhibitor of anti apoptotic protein BCL2
ABT-263	Selection inhibitor of anti apoptotic proteins BCL2 and BCL-xl
Erlotinib	Inhibitor of EGFR pathway
Everolimus	Inhibitor of mTOR pathway
Gemcitabine	Nucleoside analog inhibiting DNA replication and repair
Mitomycin C	DNA crosslinking agent
Rapamycin	Inhibitor of mTOR pathway
Sunitinib	Inhibitor of Receptor Tyrosine Kinase pathway

### Statistical approach for synergy confirmation

Once a combination of two compounds is selected, CIVO can subsequently be employed to test for synergy of those compounds. For this purpose, we model the radial response to the combination as

$$f\left(r\right)=\beta_{1}\left(r\right)+\beta_{2}\left(r\right)+\beta_{s}\left(r\right)+\alpha \left(r\right)$$

where β_1_(*r*) is the radial response induced by drug 1, β_2_(*r*) is the radial response induced by drug 2, β_*s*_(*r*) is the additional synergistic effect of the combination of both drugs, and α(*r*) is the response due to the act of injection. A value of β_*s*_(*r*) greater than zero indicates synergy.

An important consideration regarding this response model is that pharmacologically, combined responses are not characterized by such algebraic sums [8]. Typically, the algebraic sum overestimates the true additive response and can generate non-physical sums that exceed 100% response. The net result is that this model tends to underestimate synergy. For small responses (<<100%), however, an algebraic sum is a good approximation. Therefore, this approach is best applied in regions where all responses are well under 100%.

The model parameters are estimated via a linear mixed model with fixed effects β_1_(*r*), β_2_(*r*), and β_*s*_(*r*), and α(*r*) modeled as a tumor-dependent random effect. Fitting this model requires the use of four needles per tumor: one with both drugs in combination, one with drug 1 alone, one with drug 2 alone, and one with vehicle alone. Positive values of β_*s*_(*r*) with p<0.05 are taken to indicate statistically significant synergy.

### Combination index

Under certain restrictive assumptions, the model parameters estimated from the combination model above can be used to determine the combination index (CI) of Chou and Talalay [9]. The CI is defined by

$$\text{CI}=\frac{D_{1}}{{\left(D_{x}\right)}_{1}}+\frac{D_{2}}{{\left(D_{x}\right)}_{2}}$$

Where doses *D*_1_ of agent 1 and *D*_2_ of agent 2 have a combined fractional effect *x*, and (*D*_x_) _1_ and (*D*_x_) _2_, respectively, are the doses of agents 1 and 2 alone required to achieve the same fractional effect. A value of *x* equal to 50% (the “median” effect) is often targeted. A CI less than 1 indicates synergy.

By applying the median effect relationship [9] that relates effect size and dose, and assuming a first-order sigmoidal dose response relationship, the CI can be equivalently written as a function of radial distance.

$$\text{CI}\left(r\right)=\left[\frac{\beta_{1}\left(r\right)}{1-\beta_{1}\left(r\right)}+\frac{\beta_{2}\left(r\right)}{1-\beta_{2}\left(r\right)}\right]\times [\frac{1-\left(\beta_{1}\left(r\right)+\beta_{2}\left(r\right)+\beta_{s}\left(r\right)\right)}{\beta_{1}\left(r\right)+\beta_{2}\left(r\right)+\beta_{s}\left(r\right)}].$$

See Appendix A for details of the derivation.

## Results

### Multi-drug combination analysis in tumors highlights the combination of ABT-263 and Abraxane^®^

**Fig 1:**
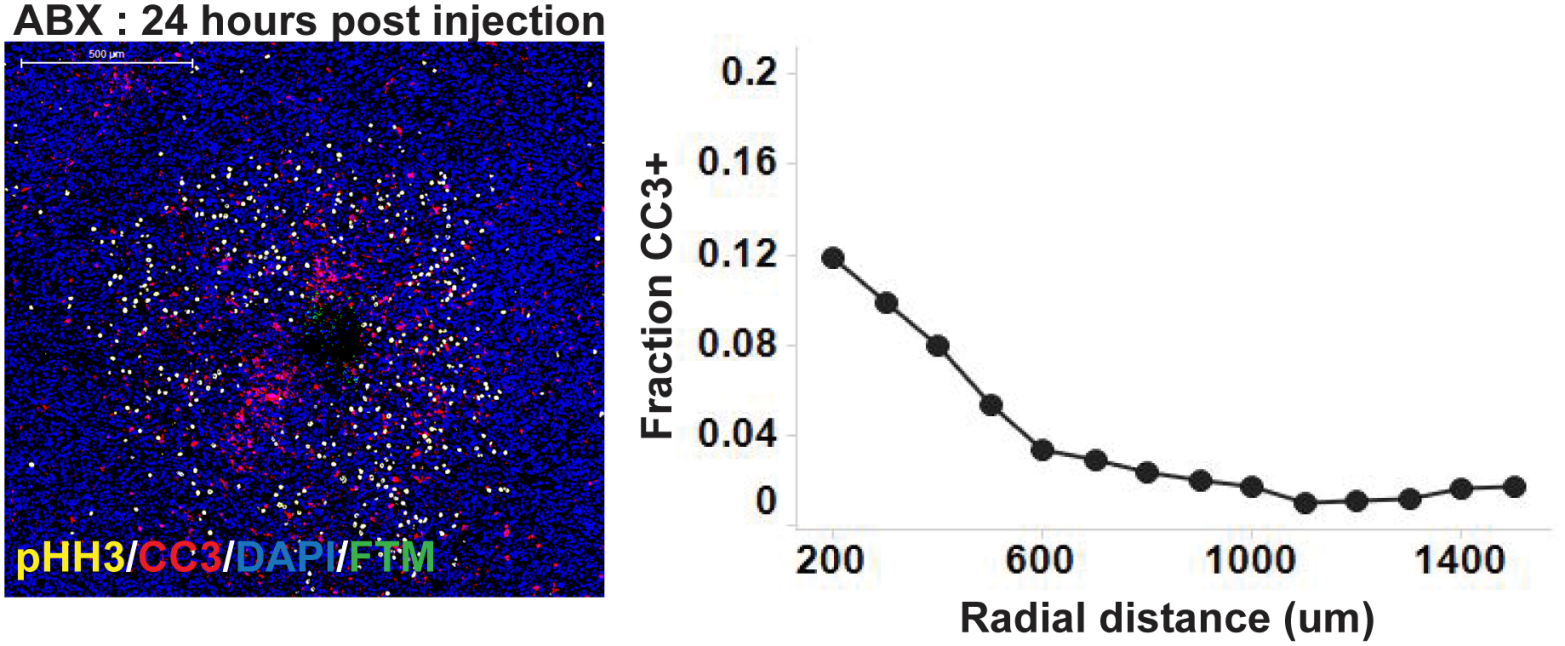
CIVO injection of Abraxane^®^ results in localized mitotic arrest but minimal tumor cell death. MiaPaCa2 tumors (n = 5) were injected using the CIVO device with Abraxane^®^ (ABX) and resected 24 hours post injection. Tissue sections were stained for phospho Histone H3 (pHH3) as a marker of mitotic arrest and cleaved caspase 3 (CC3) as a marker of apoptosis with DAPI as the counterstain. Representative image shows a single site of ABX microinjection with the fluorescent tracking marker (FTM) denoting the site of injection. Representative radial effect curve shows the fraction of CC3+ cells as a function of radial distance from the site of injection.

**Fig 2:**
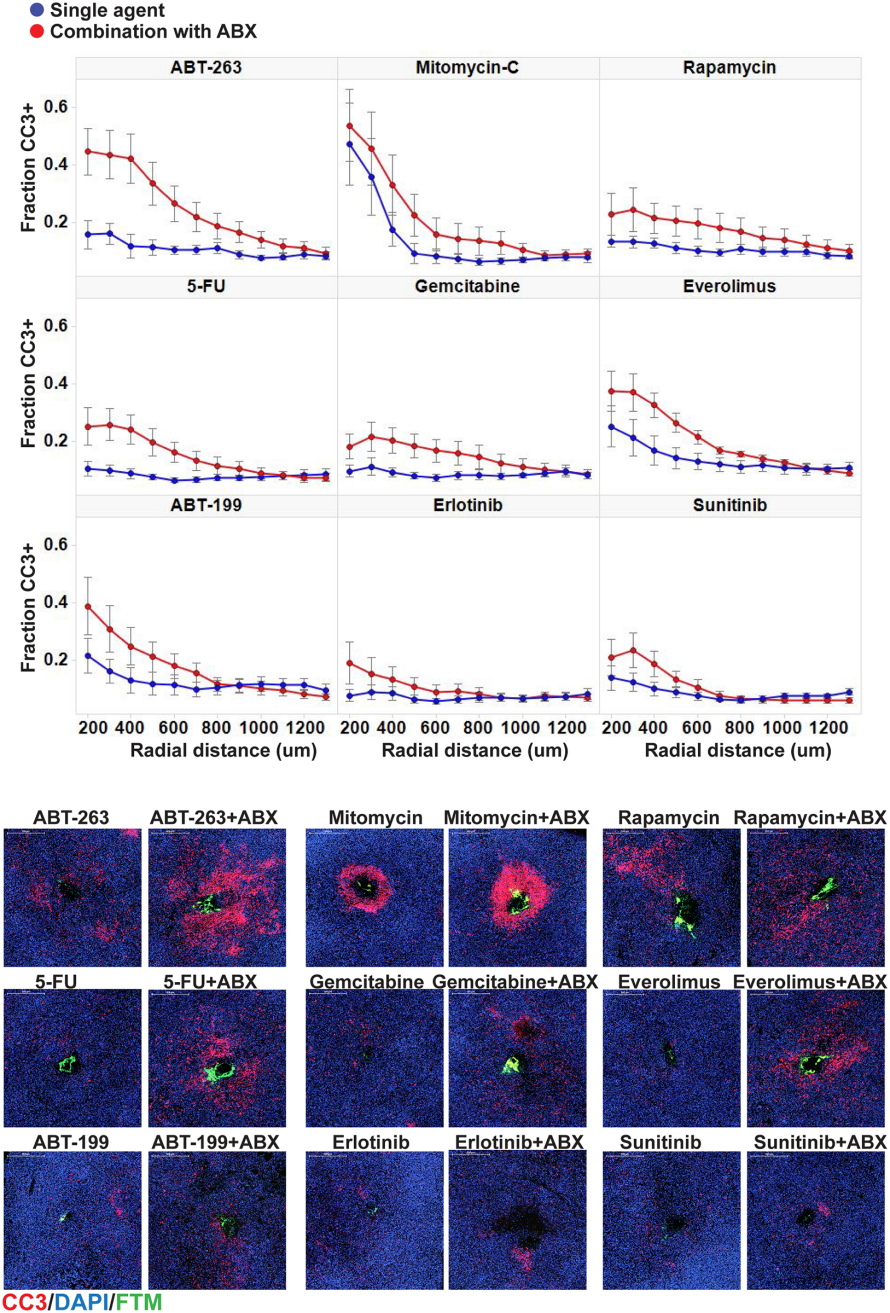
CIVO multi drug combination analysis in a xenograft model of pancreatic cancer. Using the CIVO device, MiaPaCa2 tumors were injected with single agents and combinations thereof with Abraxane^®^ (ABX) into the same tumor (n = 4 tumors per single agent/ABX combination pair) and resected 24 hours post injection. Tissue sections were stained with cleaved caspase 3 (CC3) as a marker of apoptosis with DAPI as the counterstain. Representative radial effect curves show fraction CC3+ cells as a function of radial distance from the site of injection as demarcated by FTM. Data are averaged across four tumors. Error bars denote standard error of the mean (SEM). Representative images show cytotoxic responses at injection sites of single agents and combinations thereof with ABX.

As observed previously and consistent with the ability of Abraxane^®^ to induce mitotic arrest, localized tumor exposure to Abraxane^®^ induced a substantial increase in the fraction of pHH3-postive cells surrounding the site of microinjection [10] (Fig 1). In contrast, induction of apoptosis following single agent Abraxane^®^ exposure was minimal and limited to the immediate region around the site of injection where drug exposures tend to be super-physiological. Similar to Abraxane, most of the other single agents tested induced no increase or modest increases in apoptotic tumor cell death (Fig 2). One notable exception included robust localized tumor responses to Mitomycin C, which is consistent with previous observations of responsiveness to this agent in PalB2 mutant gemcitabine resistant pancreatic cancer [11].

**Fig 3:**
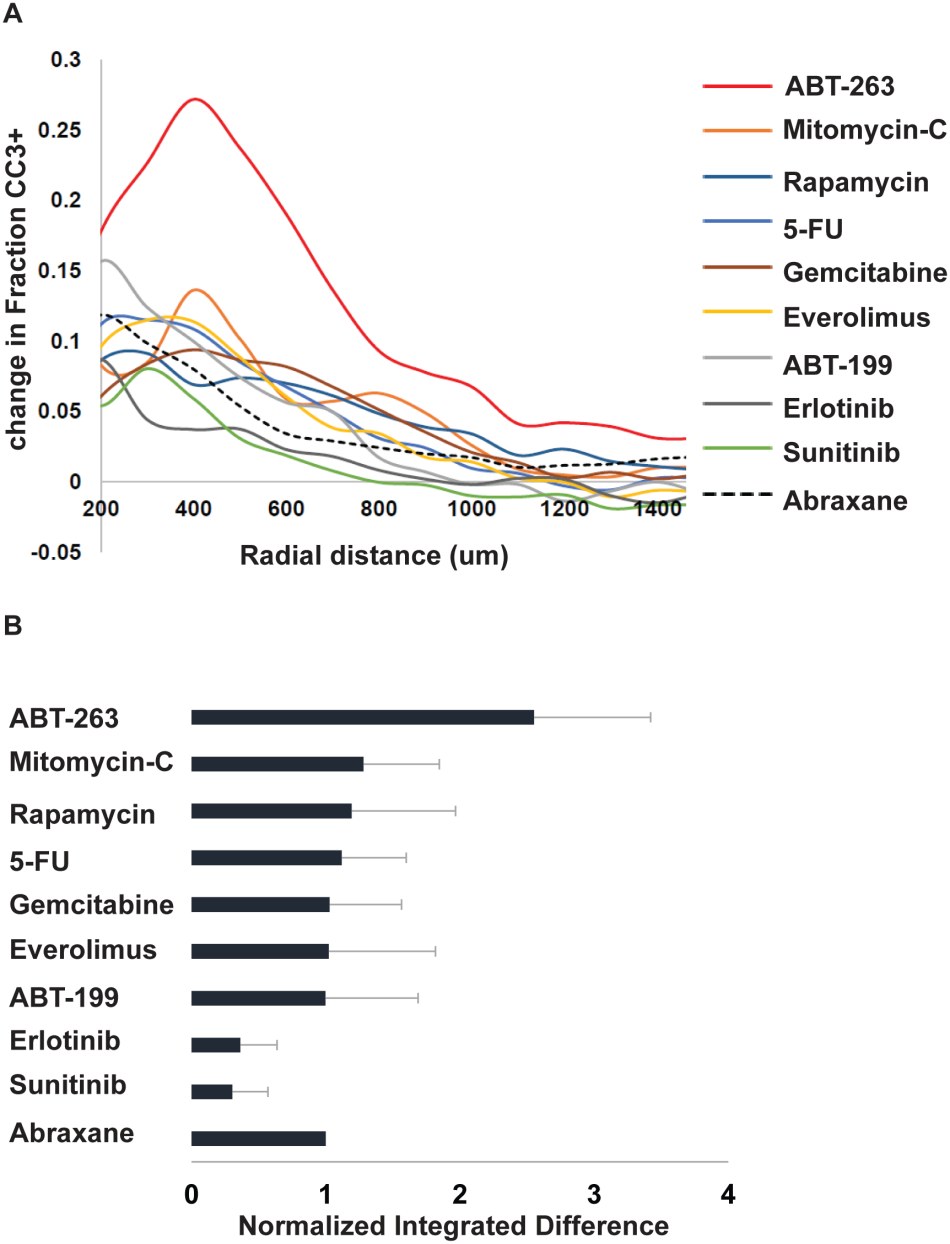
CIVO analysis identifies ABT-263 as the agent that leads to the greatest increase in cytotoxic response when combined with Abraxane. (A) Plots show the difference in fraction CC3+ cells averaged across n = 4 MiaPaCa2 tumors for each agent combined with Abraxane^®^ (ABX) versus the corresponding single agent, as a function of radial distance from the injection site. Among the curves, ABT-263 shows a greater than two-fold higher increase compared to other agents. The increases for other agents were similar to the single-agent effect of ABX alone (dashed curve) suggesting simple additivity of responses for these agents. (B) For statistical comparisons, the areas under each curve were integrated and normalized, dividing by the average area under the ABX curve. The bar chart shows estimates of these integrated differences with standard errors derived from a linear mixed effects model. Of the combinations tested, only ABT-263 plus ABX elicited a statistically significant greater apoptotic response than ABX alone (p<0.01 Wald’s test).

**Fig 4:**
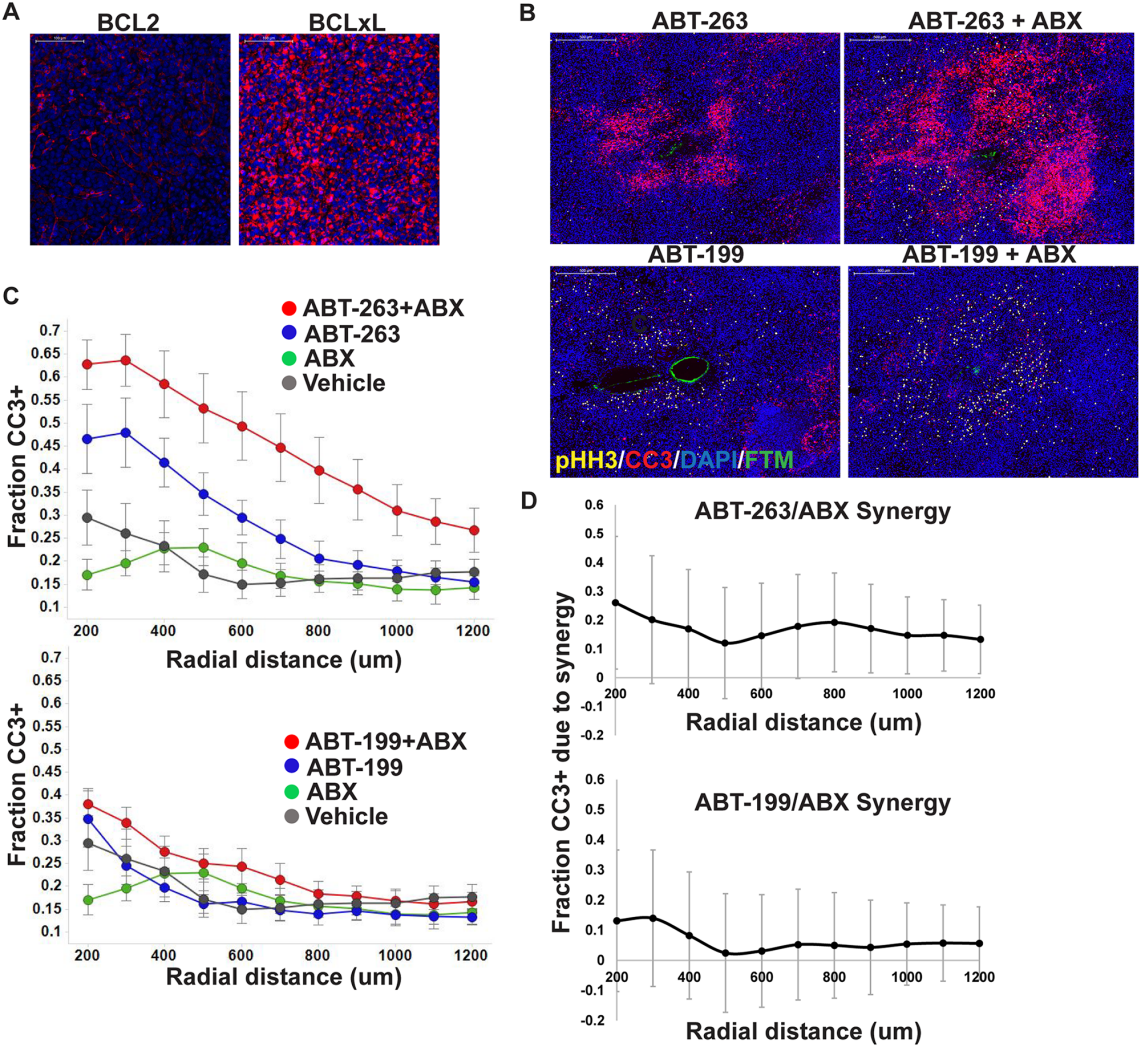
CIVO combination analysis leads to identification of a synergistic combination between ABT-263 and Abraxane^®^ but not with other selective BCL2 inhibitor, ABT-199. (A Representative images show immunohistochemical staining of target proteins BCL2 (ABT-263 and ABT-199) and BCLxL (ABT-263 only) in MiaPaCa2 xenograft tumors. (B) Using the CIVO device, MiaPaCa2 tumors were injected with vehicle, ABT-199, ABX, ABT-199+ABX or vehicle, ABT-263, ABX, ABT-263+ABX into each of 5 tumors per combination set and resected 24 hours post injection. Tissue sections were stained with cleaved caspase 3 (CC3) as a marker of apoptosis, phospho Histone H3 (pHH3) as a marker of mitotic arrest with DAPI as the counterstain. FTM demarcates the site of injection. Representative images show localized cytotoxic responses induced by ABT-263, ABT-199 and combinations thereof with ABX. (C) Plots show fraction CC3+ cells as a function of radial distance from the site of injection. Data are averaged across five tumors. Error bars denote SEM. (D) A linear mixed effects model was used to estimate the difference (β_*s*_(*r*)) between the response due to each combination versus the sum of the individual responses, which were plotted with 95% confidence intervals for each radial distance. Only ABT-263 showed statistically significant synergy (P<0.05; confidence intervals do not intersect 0) at several radial distances.

An initial investigation of several rationally selected agents combined with Abraxane^®^ revealed that the addition of Abraxane^®^ induced a modest increase in apoptosis when combined with each of the single agents tested (Fig 2). However, the combination of Abraxane^®^ with the BCL2/BCLxl inhibitor ABT-263 notably surpassed the others with the highest increase in regional CC3 signal (Fig 3). Concomitantly, the increase in CC3+ cell fraction with the drug combination was accompanied by a reduction in the pHH3+ cell fraction as compared to levels induced by Abraxane^®^ alone (S2 Fig). Interestingly, the more selective BCL2 inhibitor, ABT-199 showed an unremarkable increase in apoptotic response when combined with Abraxane^®^. Upon further investigation, this differential sensitivity was found to correlate with elevated expression of BCLxL compared to BCL2 in MiaPaCa2 tumor cells (Fig 4A) similar to other cancers of epithelial origin [12–14].

### CIVO drug combination analysis leads to confirmation of a synergistic interaction between ABT-263 and Abraxane^®^

To further validate the enhancement of Abraxane^®^-induced apoptosis and confirm the apparent mechanistic differences between ABT-199 and ABT-263, both BCL2 inhibitors were selected for quantitative synergy analysis with Abraxane^®^. MiaPaCa2 tumors were each injected with Abraxane^®^, ABT-199 and ABT-263 alone, Abraxane^®^ in combination with each of ABT-199 and ABT-263, and a vehicle control. Consistent with the results from our initial interrogation, localized exposure to Abraxane^®^ resulted in enrichment of cells arrested in mitosis but only a limited increase in cells undergoing apoptosis, while exposure to ABT-199 alone did not induce a notable single agent anti-tumor response. Interestingly in this validation experiment, some of the tumor sites subjected to ABT-263 exposure exhibited significant enrichment of CC3+ cells. Importantly, the combination of Abraxane^®^ and ABT-263 induced significantly higher levels of CC3+ apoptotic cells compared to either drug alone consistent with findings from the previous experiment (Fig 4B and 4C). This included substantial enrichment of CC3+ cells at radial distances (>900 micron) where neither Abraxane^®^ nor ABT-263 induced anti-tumor effects as single agents. Application of our statistical model of synergy indicated that the synergy term β_*s*_(*r*) was significantly higher than 0 at a wide range of radial distances extending out to at least 1200 μm for the combination of Abraxane^®^ and ABT-263 (Fig 4D). Across all radial distances, the magnitude of this increase was near 0.2, indicating that 20% of the cell death, in absolute terms, could not be accounted for by a simple sum of the individual agent responses. The combination index (CI) was found to be less than or equal to 0.5 at all radial distances, indicating strong synergy. Consistent with a requirement for BCL-xL inhibition to achieve synergistic anti-tumor effects in combination with taxanes in tumors of epithelial origin [12–14], the combination of Abraxane^®^ and ABT-199, generally elicited only a slightly elevated response compared to either drug alone and β_*s*_(*r*) was not significantly different from 0 for the combination of Abraxane^®^ and ABT-199 at any of the radial distances tested (Fig 4).

### Tumor growth inhibition with systemically delivered Abraxane^®^ and ABT-263 confirms CIVO prediction

**Fig 5:**
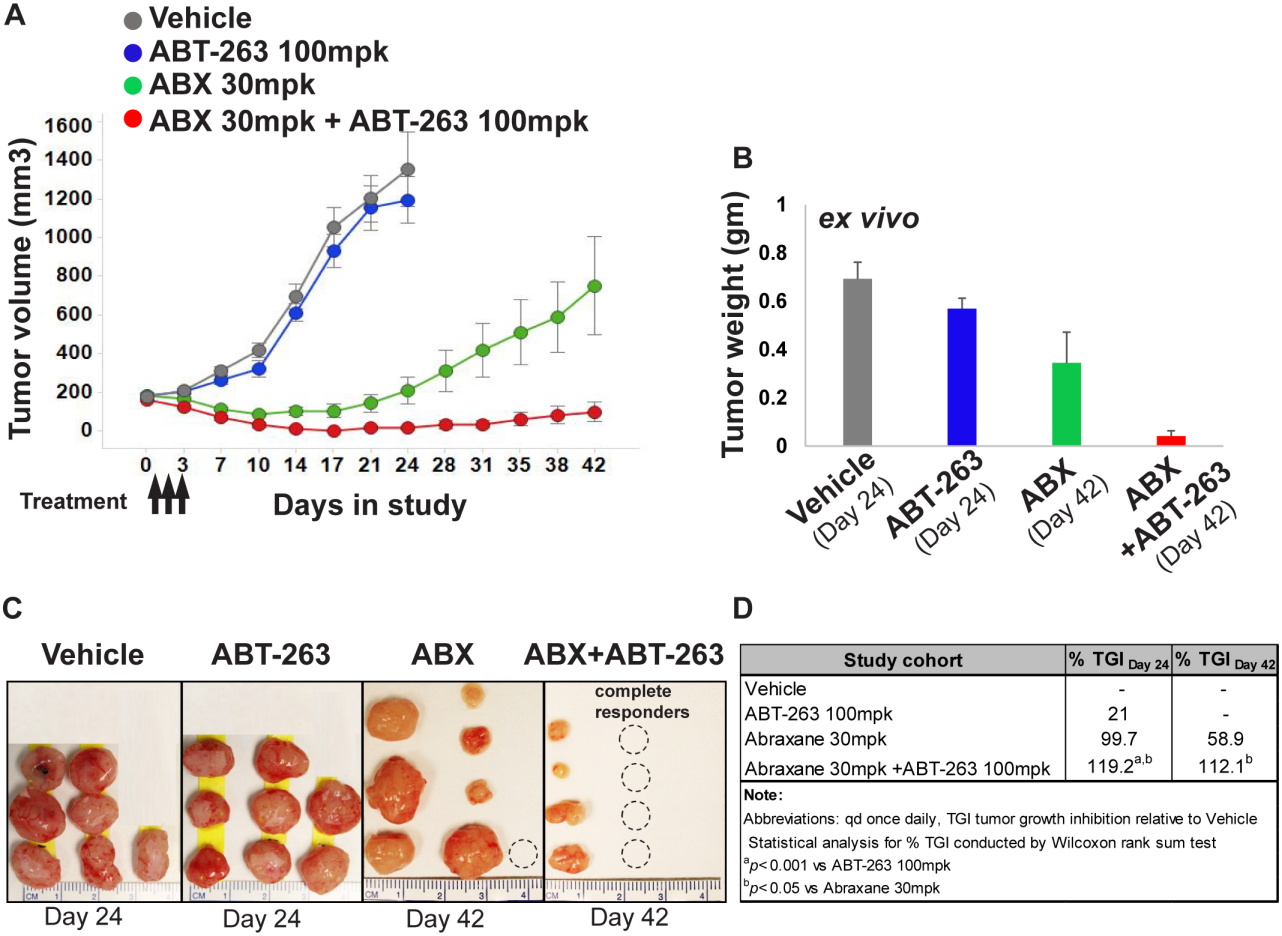
Abraxane^®^ combined with ABT-263 leads to 50% complete response and durable tumor remission in a pancreatic cancer model. (A) MiaPaCa2 xenografted mice (n = 8 per treatment cohort) were treated systemically with vehicle (control), ABT 263 100 mpk PO Days 1–3, Abraxane^®^ (ABX) 30 mpk IV Days 1–3 or combination of ABT 263 and ABX using the same dosing regimen as the single agents. Drug efficacy with respect to vehicle was assessed in all arms via tumor volume measurements and (B) via *ex vivo* tumor weight measurements. *p* < 0.05 (two sided Student’s t-test) for ABX+ABT-263 vs ABT263 or ABX. Data are averaged across all tumors in the respective cohorts. Error bars represent SEM. (C) Representative *ex vivo* images of tumors from each treatment arm on Day 24 and Day 42 (D) Tumor Growth Inhibition (TGI) index was calculated at Day 24 when vehicle and ABT-263 arms met censoring criteria (see Methods) and on Day 42 for the remaining ABX and ABX+ABT-263 arms. *p* values for TGI comparisons were calculated using the Wilcoxon Rank Sum test.

Using the CIVO platform, we have previously shown that localized tumor responses to single agent chemotherapeutics and targeted drugs are predictive of long term systemic efficacy [5]. To determine whether a localized synergistic response to a transient micro-dosed drug combination translates to long term efficacy, a systemic study was conducted with Abraxane^®^ and ABT-263 in the same model used in the CIVO analysis. MiaPaCa2 tumors (average volume ~200mm3) were systemically treated with Abraxane, ABT-263 or a combination thereof, based on dosing regimens previously described for both drugs [12, 15–18]. Consistent with the increased region of apoptotic cells observed upon localized co-exposure to Abraxane^®^ and ABT-263, while Abraxane^®^ showed prominent single agent efficacy compared to vehicle, combining it with ABT-263 even at the lowest Abraxane^®^ dose yielded a significantly superior growth inhibitory effect and durable tumor remission (Fig 5 and S3 Fig). Moreover, 50% (4/8) of the cohort treated with 30 mpk ABX + 100 mpk ABT-263 underwent complete remission as compared to 12.5% (1/8) in the 30 mpk Abraxane^®^ single agent arm and none (0/8) in the ABT-263 single agent arm. Lower doses of Abraxane^®^ (10 and 20 mpk) combined with ABT-263 100 mpk also led to 25% (2/8) complete remission (S3 Fig). All treatments were well-tolerated with no overt signs of toxicity or body weight changes (S4 Fig). Therefore, as we have demonstrated previously with single agent studies, CIVO analysis of drug synergy accurately predicted the outcome of the more resource intensive systemic study showing clearly greater anti-tumor responses induced by the ABX/ABT-263 combination than those induced by either drug alone.

## Discussion

Advances in our collective understanding of the molecular mechanisms that promote cancer cell survival and the clinical limitations of most single agent therapies for treating solid malignancies has prompted academic and industry wide efforts to identify effective combination therapies. These efforts are beginning to significantly improve outcomes for many patients with notoriously difficult to treat cancers. For instance the combination of Abraxane^®^ and Gemcitabine, moderately extends median survival of patients with pancreatic cancer [18]. Unfortunately therapeutic responses to this, and several other combinations of drugs are typically short lived, and patients ultimately succumb to their disease. Thus there is a clear need to identify novel effective drug combinations that can be used to treat solid tumors such as pancreatic cancer and there are several groups active in this endeavor [19–23]. While a number of methods exist to identify synergistic drug combinations in the preclinical setting, our ultimate goal is to develop an approach that is not limited to the laboratory, but could feasibly be employed to assess drug combinations directly in a cancer patient’s tumor. Here we demonstrate several key steps towards that capability.

In this study we present a novel application of the CIVO microinjection platform that enables rapid identification and quantitative evaluation of drug combinations *in vivo* across diverse classes of anti-cancer chemotherapeutics and targeted agents. First, we demonstrated the ability to compare responses across a panel of combinations to identify the most promising candidate(s) in live tumors. This led us to select ABT-263 as a target of interest for combination with Abraxane^®^ in tumors derived from the MiaPaCa2 pancreatic cancer cell line. Second, we demonstrated the ability to identify and statistically validate the presence of synergy in response to a specific combination. This allowed us to confirm that the combination response of ABT-263 and Abraxane^®^ exhibits synergy in the same sense as this term is used to describe drug interactions in cell-based assays and with similar precision, bringing this level of rigor for the first time into the *in vivo* realm. We also demonstrated the absence of drug synergy using ABT-199, a closely related derivative of ABT-263, in combination with Abraxane^®^. The responses correlated with the expression of the respective target proteins thereby demonstrating the capability of the CIVO platform to identify context-specific drug combinations. Finally, key to demonstrating the utility of our approach, the synergistic interaction between Abraxane^®^ and ABT-263 observed upon co-localized microinjection, was borne out in a traditional 4-arm preclinical drug combination study where tumor growth inhibition was measured over time following systemic drug treatment. This suggests that the localized anti-tumor drug combination responses, measured within days of drug microinjection by histological biomarker analysis, are meaningful in terms of accurately predicting the results of longer term studies where effects of systemically administered drugs can be assessed by more traditional measures of tumor size and progression. Our findings are consistent with previously published data that demonstrate enhanced efficacy in other cancer models when ABT-263 is paired with a taxane [12, 15, 24, 25]. Therefore, one important capability of CIVO is expedited combination screening *in vivo*, enabling efficient prioritization of drug combinations that merit further investigation in more resource intensive studies, thereby potentially greatly reducing the timeframe from effective drug combination identification to clinical trials.

Alternative strategies for assessing anti-cancer drug combinations *in vivo* have been proposed, notably using the popular Chou Talalay approach for combination analysis [9]. To use the Chou Talalay approach in xenografted tumors, however, 50–80 mice are suggested for the assessment of a single combination of agents [26]. In contrast, here we assessed 9 different combinations and further explored 2 of those combinations for synergy using only 19 mice in total. This assessment of drug combination efficacy was executed in less than two weeks from time of drug injection to final data analysis. The efficiency of the CIVO approach derives from several key aspects of the platform. First, CIVO enables multiple agents or combinations to be tested in the same tumor. Second, the pairing of combination injections and single agent injections in the same tumors is statistically more powerful than comparing responses in separate tumors. Third, CIVO injections induce a spatially varying concentration that enables individual responses to be probed for dosages ranging from super-physiological (within a few hundred μm of the injection) to the threshold of response (>1mm from the injection). Together these advantages make large scale screening approaches possible without requiring a prohibitive number of tumors. Indeed, our previous demonstration of *in vivo* drug screening capability [5] suggests that for preclinical drug development programs scale-up of our method to screen up to hundreds of combinations of interest across a panel of cancer models is possible.

The combination of Abraxane and ABT-263 may represent a novel therapy, with a demonstrated mechanistic basis for anti-tumor synergy, for pancreatic cancer patients who have failed gemcitabine-based treatments. An interesting observation, as revealed by the CIVO study, was the reciprocal relationship between localized CC3 and pHH3 positive cell subpopulations in response to Abraxane^®^ versus the combination of Abraxane^®^ and ABT-263. The data is supportive of a previously described model proposing a mechanism for the synergistic anti-tumor activity induced by ABT-263 and taxanes [12]. Three potential fates for cancer cells arrested in mitosis following taxane exposure are proposed, two of which ultimately lead to tumor cell survival (mitotic slippage and delayed re-entry into the cell cycle), while the third results in apoptotic cell death. Our data showing the concomitant loss of pHH3+ cells and gain in CC3+ cells in Abraxane/ABT-263 co-microinjected regions is consistent with the proposal that inhibition of BCL2/BCLxl drives cells towards the apoptotic fate ultimately leading to tumor regression. This is the first direct *in vivo* demonstration in support of this model of synergy [12]. Consistent with an increase in drug induced apoptosis leading to a more durable tumor regression in the systemic study, 4 out of 8 mice treated with one cycle of the Abraxane/ABT-263 combination showed no evidence of tumor tissue weeks following the last dose of drug. The well-documented adverse effects including grade 4 thrombocytopenia observed in clinical trials investigating ABT-263 are likely to preclude treatment with dosing regimens capable of achieving sufficient single agent exposures to induce anti-tumor response with an acceptable therapeutic index. Our data, consistent with findings from other laboratories, suggests that the therapeutic potential of ABT-263 can be realized if combined with drugs such as Abraxane^®^ where clear anti-tumor synergy is observed. This may allow for lower, more tolerable doses of ABT-263 to be administered to patients so that anti-tumor effects are maintained under a reduced requirement for high tumor drug concentrations, thereby decreasing unintended impact on normal cell compartments.

While the studies presented here represent critical steps forward, ultimately the value of CIVO technology as applied to drug combinations lies in direct investigation of combination efficacy in the human cancer clinic. It is reasonable to expect that localized responses to direct drug microinjection into solid tumors, while the tumor mass is still in the patient, will capture elements of tumor biology and tumor drug interactions not captured by advanced preclinical models. For instance, patient-derived xenografts preserve some of the complex biology observed in human tumors, and a recent study demonstrated the feasibility of using such models in a high throughput screen format [27]. However, the use of these models is still limited to studies in the context of immune-deficient hosts and thus ultimately lacks potentially important interactions between tumor cells, drugs, and the host’s immune system. Our early studies in the canine and human oncology clinics have shown the impact of non-tumor cell components on drug exposures ([5] and unpublished work). These have included but are not limited to stromal barriers impacting drug penetration into the tumor, and drug-induced infiltration of immune cells around the sites of drug injection. Further clinical investigation and key correlation studies with patient outcome will be necessary to truly determine the ultimate utility of this approach as a toxicity-sparing method to predict drug combination efficacy for both development of such combinations and for a personalized medicine approach to selection of effective treatments on a per patient basis.

In summary, we have presented a novel method for diagnostic assessment of drug combinations in living tumors using the CIVO technology. This approach can currently be applied to medium throughput drug combination screens that should significantly reduce the preclinical costs and time to validation for translation of drug combinations into the clinic. More importantly, CIVO technology holds the potential for use in toxicity-sparing studies directly in human patients to segregate responders from non-responders enabling improved clinical trial stratification.

## Appendix A: Derivation of combination index equation

The median effect relationship for a first-order dose response relationship [9] is

$$\frac{D}{\left(D_{50}\right)}=\frac{\beta }{1-\beta }$$

where *D* is any dose, β is the fractional effect at that dose, and *D*_50_ is the dose for which β = 50%. More generally, we can write this in terms of a dose *D*_*x*_ that produces a fractional response *x* as

$$\frac{D}{\left(D_{x}\right)}=\frac{D}{\left(D_{50}\right)}\frac{\left(D_{50}\right)}{\left(D_{x}\right)}=\left[\frac{\beta }{1-\beta }\right]\times [\frac{1-x}{x}].$$

Thus, the CI defined by

$$\text{CI}=\frac{D_{1}}{{\left(D_{x}\right)}_{1}}+\frac{D_{2}}{{\left(D_{x}\right)}_{2}}$$

can equivalently be written

$$\text{CI}=\left[\frac{\beta_{1}}{1-\beta_{1}}+\frac{\beta_{2}}{1-\beta_{2}}\right]\times [\frac{1-x}{x}].$$

In this equation, *x* is the fractional effect of the combination of agents, given at a specific radial distance in our model by

$$x=\beta_{1}\left(r\right)+\beta_{2}\left(r\right)+\beta_{s}\left(r\right)$$

(Note that α(*r*) is not included in this equation, because it captures effects unrelated to the drug). Thus, plugging this in for *x* and writing the complete equation as a function of radial distance gives

$$\text{CI}\left(r\right)=\left[\frac{\beta_{1}\left(r\right)}{1-\beta_{1}\left(r\right)}+\frac{\beta_{2}\left(r\right)}{1-\beta_{2}\left(r\right)}\right]\times [\frac{1-\left(\beta_{1}\left(r\right)+\beta_{2}\left(r\right)+\beta_{s}\left(r\right)\right)}{\beta_{1}\left(r\right)+\beta_{2}\left(r\right)+\beta_{s}\left(r\right)}].$$

## Supporting Information

S1 Fig Immunohistochemical characterization of the microenvironment of a representative MiaPaCa2 xenograft tumor shows heterogeneous composition of the stroma. Representative image from a MiaPaCa2 xenograft tumor shows Masson Trichrome staining for stromal collagen and immunohistochemical staining for markers of hypoxia (GLUT1), blood vessel endothelial cells (CD31), α-smooth muscle actin (αSMA) and infiltrating macrophages (S100A9) in the tumor stroma.
(TIF)

S2 Fig ABT-263 reduces G2/M arrested cells induced by Abraxane^®^ and increases apoptotic cell fraction. Radial effect curves show fraction pHH3+ and CC3+ cells as a function of radial distance from the site of injection. Data are averaged across four tumors. Error bars denote SEM.
(TIF)

S3 Fig Abraxane^®^ at doses lower than the clinically relevant dose combined with ABT-263 also leads to tumor regression and remission in the MiaPaCa2 pancreatic cancer model. (A-B) MiaPaCa2 xenografted mice (n = 8 per treatment cohort) were treated systemically with vehicle (control), ABT 263 100 mpk PO Days 1–3, Abraxane^®^ (ABX) 10 or 20 mpk IV Days 1–3 or combination of ABT 263 and Abraxane^®^ using the same dosing regimen as the single agents. Drug efficacy with respect to vehicle was assessed in all arms via tumor volume measurements when tumors met censoring criteria (See Methods). Data are averaged across all tumors in the respective cohorts. Error bars represent SEM. (C) Representative *ex vivo* images of tumors from each treatment arm on Day 24 and Day 35.
(TIF)

S4 Fig Systemic administration of ABX, ABT-263 or combinations thereof, did not induce any significant body weight changes at any dose level. Plots represent body weight of mice recorded over time during treatment (Days 1–3) and 12 days after cessation of treatment across all treatment groups. Data are averaged across all tumors in the respective cohorts. Error bars represent SEM.
(TIF)
