# Supplementary figures and images for: A Platform for Rapid, Quantitative Assessment of Multiple Drug Combinations Simultaneously in Solid Tumors In Vivo
Source: PLoS One. 2016 Jun 30;11(6):e0158617. doi: 10.1371/journal.pone.0158617 (PMC4928803; doi:10.1371/journal.pone.0158617)

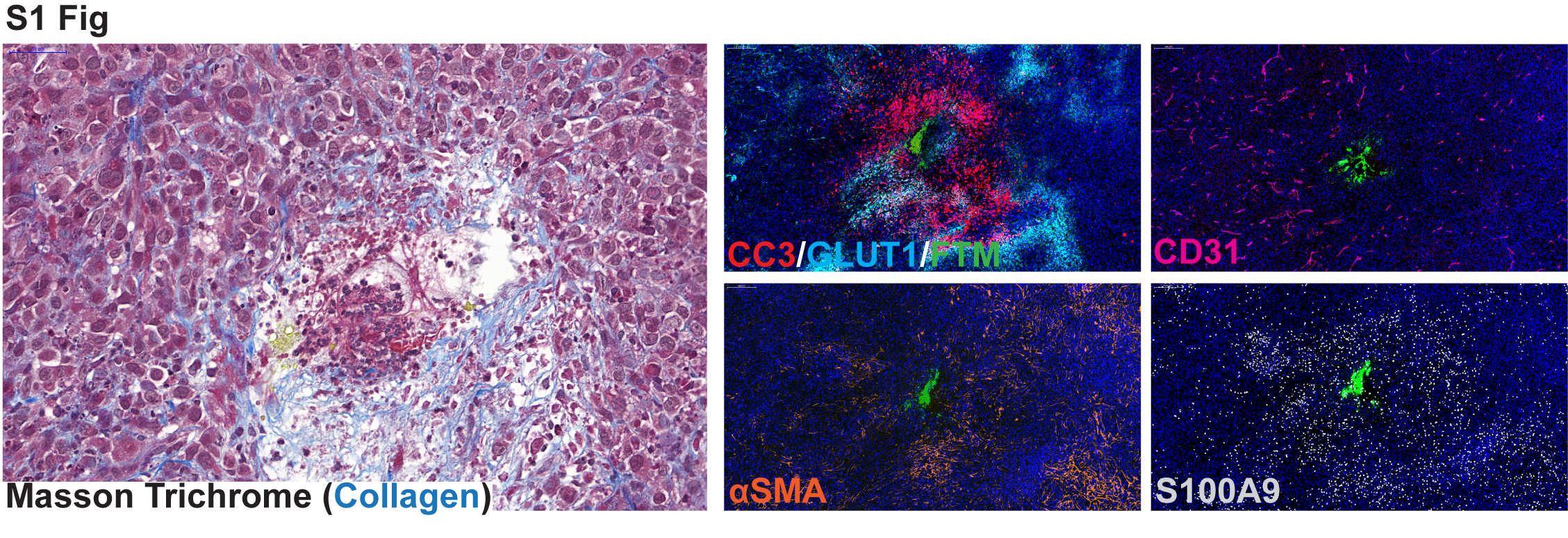

Supplement: S1 Fig — Representative image from a MiaPaCa2 xenograft tumor shows Masson Trichrome staining for stromal collagen and immunohistochemical staining for markers of hypoxia (GLUT1), blood vessel endothelial cells (CD31), α-smooth muscle actin (αSMA) and infiltrating macrophages (S100A9) in the tumor stroma. (TIF) [file pone.0158617.s001.tif]

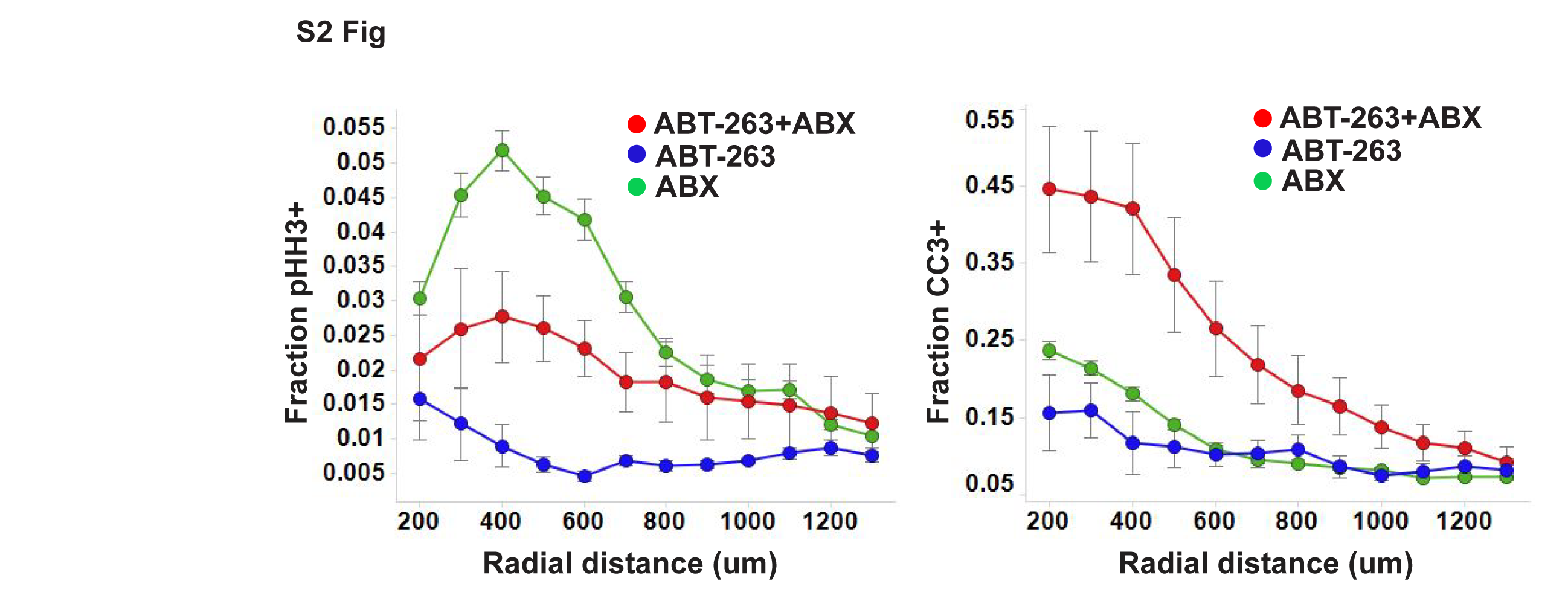

Supplement: S2 Fig — Radial effect curves show fraction pHH3+ and CC3+ cells as a function of radial distance from the site of injection. Data are averaged across four tumors. Error bars denote SEM. (TIF) [file pone.0158617.s002.tif]

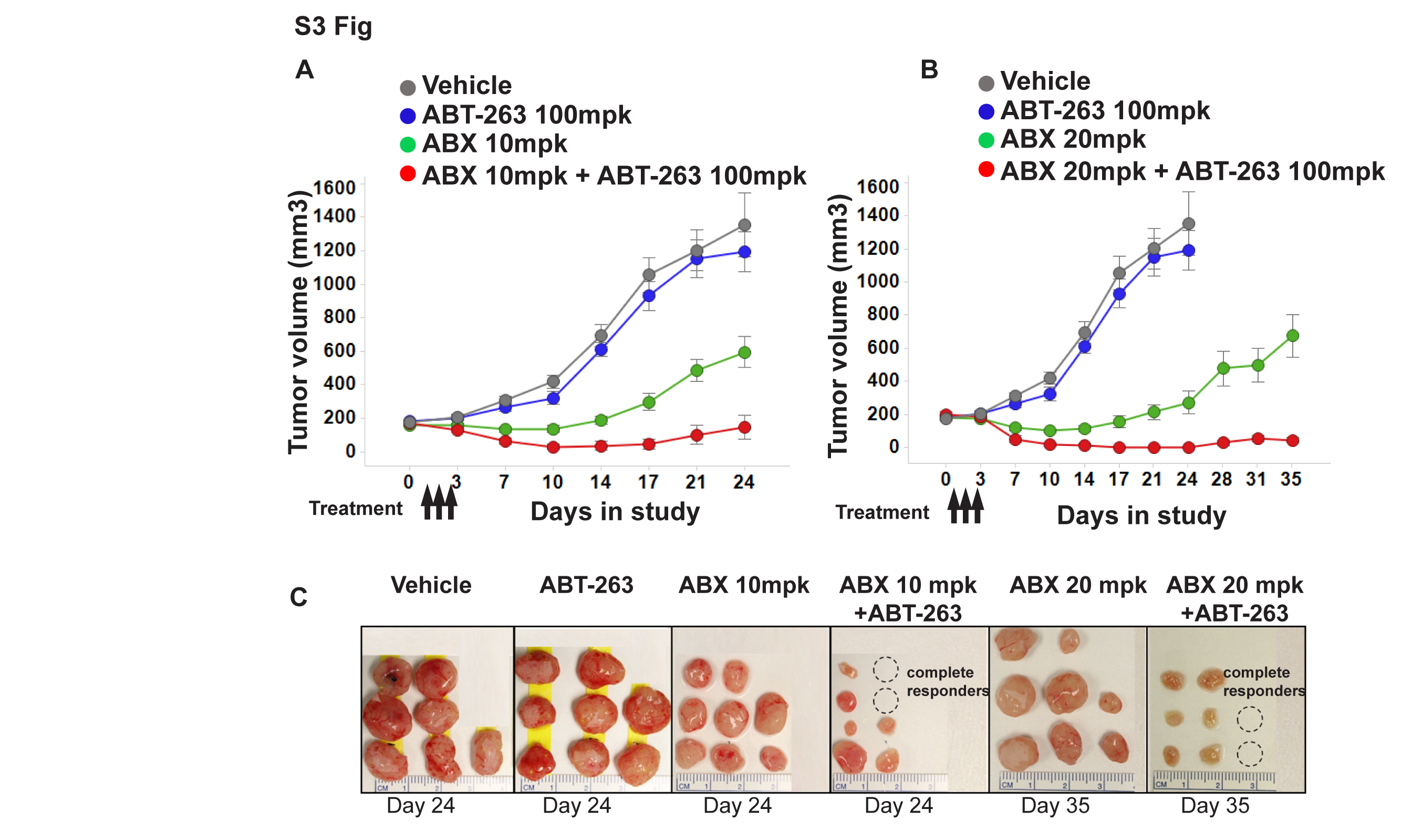

Supplement: S3 Fig — (A-B) MiaPaCa2 xenografted mice (n = 8 per treatment cohort) were treated systemically with vehicle (control), ABT 263 100 mpk PO Days 1–3, Abraxane® (ABX) 10 or 20 mpk IV Days 1–3 or combination of ABT 263 and Abraxane® using the same dosing regimen as the single agents. Drug efficacy with respect to vehicle was assessed in all arms via tumor volume measurements when tumors met censoring criteria (See Methods). Data are averaged across all tumors in the respective cohorts. Error bars represent SEM. (C) Representative ex vivo images of tumors from each treatment arm on Day 24 and Day 35. (TIF) [file pone.0158617.s003.tif]

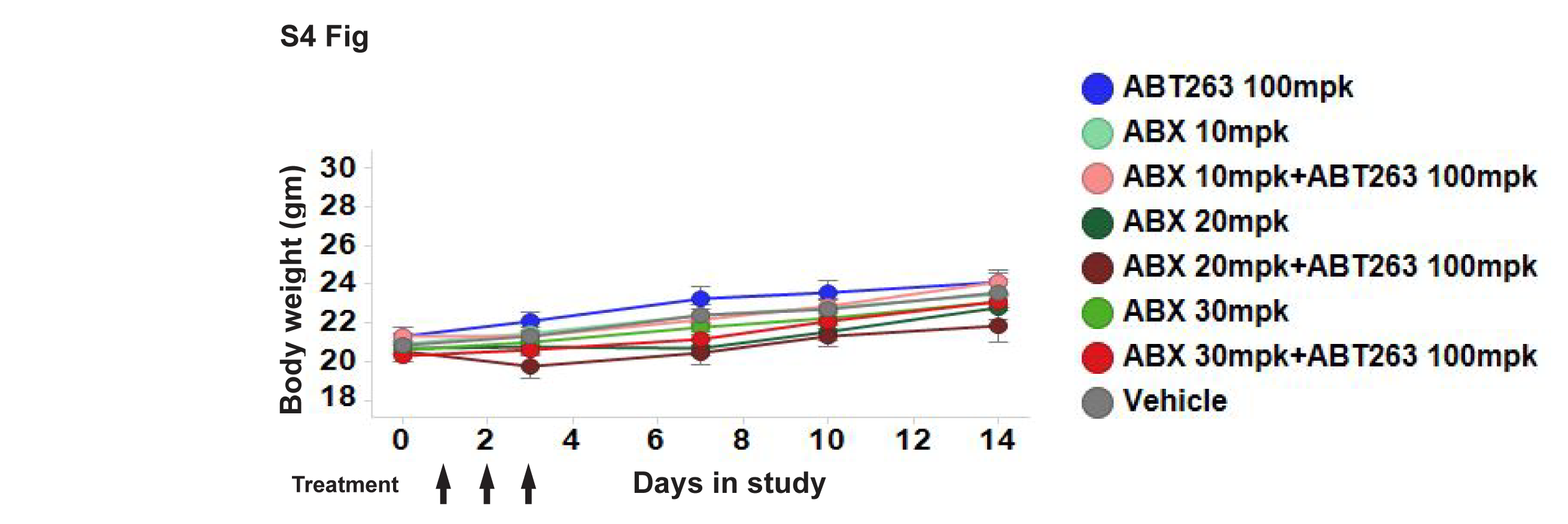

Supplement: S4 Fig — Plots represent body weight of mice recorded over time during treatment (Days 1–3) and 12 days after cessation of treatment across all treatment groups. Data are averaged across all tumors in the respective cohorts. Error bars represent SEM. (TIF) [file pone.0158617.s004.tif]
